# Regulatory effects of root pruning on leaf nutrients, photosynthesis, and growth of trees in a closed-canopy poplar plantation

**DOI:** 10.1371/journal.pone.0197515

**Published:** 2018-05-21

**Authors:** Da-wei Jing, Zhen-yu Du, Ming-you Wang, Qing-hua Wang, Hai-lin Ma, Fang-chun Liu, Bing-yao Ma, Yu-feng Dong

**Affiliations:** 1 College of Resources Environment and Planning, Dezhou University. Dezhou, Shandong, China; 2 Institute of Resource and Environment, Shandong Academy of Forestry, Jinan, China; 3 College of Ecology and Garden Architecture, Dezhou University, Dezhou, Shandong, China; Institute of medical research and medicinal plant studies, CAMEROON

## Abstract

A plantation of 5-year-old poplar *Populus × euramericana cv*. ‘Neva’ was used to study the regulatory effects of root pruning on nutrients, photosynthetic characteristics, and water-use efficiency (WUE) of leaves and growth rates of diameter at breast height (DBH; 1.3 m), tree height, and volume. Six root-pruning treatments were conducted with different combinations of intensity (at a distance of six, eight or ten times DBH from the trunk) and orientation (on two or four sides of the trees). Results showed that the N, P, K, photosynthetic rate, transpiration rate, and stomatal conductance of leaves were all significantly decreased by root pruning over the initial period following root pruning (30 days), but increased in the subsequent investigations. The values of the above indexes peaked in 8–2 treatment (i.e., eight times DBH distance on two sides). The leaf WUE in 8–2 treatment, and average growth rates of DBH, tree height and volume, were the highest among all treatments within 3 years of root pruning. The results indicated that the root pruning based on the appropriate selection of intensity and orientation had significant positive effects on leaf nutrients, photosynthesis, and growth of trees in a closed-canopy poplar plantation.

## Introduction

Compared with other tree species, poplar has many characteristics that make it suitable for cultivating in plantations, enabling the production of large quantities of wood in short periods of time. These features consist of rapid growth, adaptability to diverse environmental conditions, and suitability for different silvicultural systems [1–3]. In China, poplars are usually cultivated at a density of 830–1100 trees per ha. With the increase of growth time, the lateral roots of poplar gradually expand and inevitably intermingle with those of neighboring trees after canopy closure. Interweaved roots inhibit the growth of poplar, and is beneficial to the spread of pests and diseases, and thus, significantly reduce root vitality during this period [4–5]. In addition, the amount of fine roots decreased when poplars were at the stage of canopy closing, thereby significantly suppressing the ability of the poplar root system to absorb water and nutrients [5–7]. Therefore, how to improve root system vitality in poplar closed-canopy plantations is particularly important for tree growth and, thus, the economic return of such plantations.

Root pruning is a common technique that can reduce the vegetative growth of fruit trees [8–9]. It not only assists in dwarfing, but also stimulates the emergence of new roots necessary to sustain growth [10]. Specifically, root pruning destroys the old growth balances of trees and changes their assimilation abilities, nutrient distributions, and hormone levels. The combined action of these elements can cause different effects on growth and fruit quality [9]. Root pruning is also used in poplar tree transplanting. The root system is trimmed to a manageable size (usually to a diameter of approximately 20 cm) after lifting in preparation for storage and planting the following spring [11]. Recent studies of Chinese winter jujube (*Ziziphus jujuba* Mill.) found that root pruning significantly stimulated the germination of a large number of new roots at the incision site, increased root vitality, and expanded the absorption area of the root, thereby significantly improving the nutrition conditions and photosynthetic characteristics of the leaves [12]. The results of a previous study showed that root pruning of poplar can also improve rhizosphere soil fertility at the stage of canopy closing [4]. However, less information is available on the effects of root pruning on the leaf nutrients and photosynthetic characteristics of poplar trees.

Water-use efficiency (WUE), measured as the biomass produced per unit transpiration, describes the relationship between water use and plant productivity. The basic physiological definition of leaf WUE is equal to the photosynthesis: transpiration ratio, also referred to as the transpiration efficiency [13]. If leaf-level transpiration and stomatal conductance are measured simultaneously, these measurements can be used to determine leaf WUE [14–15]. Under certain conditions, promoting root growth and increasing root weight can increase yield and drought resistance [16]. However, more roots might not be favorable in arid and semiarid areas. There is evidence that breeding of wheat varieties has unknowingly increased WUE by reducing the size of the root system [17–18]. However, no research has been reported regarding the effect of changing the size of the root system on WUE of closed-canopy poplar.

Our previous research on canopy-closed poplar trees reported that removing some roots at a distance of 8 times the diameter at breast height (DBH) from trunk on both inter-row sides of the trees improved tree growth and rhizosphere soil fertility [4–5]. However, it is currently unknown whether cutting roots of poplars on four sides at a certain distance from trunk will have improved results compared with pruning roots on two sides. In addition, a previous study that pruned roots only on two sides of poplar trees reported only preliminary data, lacking further exploration of the effects of root pruning on nutrition, photosynthesis, and WUE of leaves; therefore, it will be informative to clarify the regulatory effects of root pruning on closed-canopy poplar plantations.

In this study, we investigated the effects of root pruning on leaf nutrients, photosynthetic characteristics, WUE, and growth of closed-canopy poplars. We hypothesized that root pruning would improve photosynthetic performance and increase tree growth. The objective of this research was to determine the feasibility of implementing root pruning in plantations and then to select optimal root pruning measures to increase the growth of poplar at the stage of canopy closing.

## Materials and methods

### Ethics statement

This research did not involve human or other animal subjects. For plant collections, we collected the minimum number of specimens necessary to ensure that appropriate vouchers were obtained. The field studies did not involve endangered or protected species. Permission to work in a poplar plantation located in Ertun township was obtained through a cooperative agreement between Dezhou University and Dezhou Forestry Bureau.

### Site description and plant material

The poplar plantation was located in Ertun township, Dezhou city, Shandong Province, north China (36°24'N latitude, 115°45'E longitude), which has a warm temperate zone continental monsoon climate with four distinct seasons, an annual average temperature of 14°C, and average annual rainfall amount of 650–700 mm. The amounts of available nitrogen (N), phosphorus (P), and potassium (K) in the soil were 35.02, 14.21 and 86.17 mg^.^kg^−1^, respectively, and the organic matter content was 11.36 g^.^kg^−1^ [19]. Soil pH was 8.56 (1:2.5 soil/water suspension). N, P_2_O_5_, and K_2_O had been carried out annually at rates of 205.36, 70.62, and 58.70 kg.ha^−1^, respectively. The fertilizers included urea, ammonium phosphate, and potassium chloride. The poplar ‘I-107’ (*Populus × euramericana cv*. ‘Neva’) had been planted 5 years previously at a spacing of 4 × 3 m. The experimental trees were uniform, and the average tree height and DBH (1.3 m) were 12.26 m and 11.25 cm, respectively. These trees are carefully managed and grown as short rotation (7–8 years) poplar mainly for pulpwood production.

### Experimental design and root pruning treatments

The experiment involved a randomized complete block design with seven treatments of three replications each. A total 21 plots was established and each replication of every treatment contained a plot with 30 trees arrayed in five rows, but only the innermost 12 trees, which were identical to the plot mean, were used for detailed measurements. Shortly after the leaves had fully developed, seven treatments were applied to the poplar trees at the beginning of the growing season on April 18^th^, 2015 and the root-pruning treatments were carried out once during the experimental period. The treatments were as follows: (1) CK, the control, with no root pruning; (2) 6–2, removing the root system at a distance six times the DBH from the trunk (74.10 cm) on both inter-row sides of each poplar; (3) 6–4, removing the root system at a distance of six times the DBH from the trunk (74.10 cm) on four sides of each poplar; (4) 8–2, removing the root system at a distance of eight times the DBH from the trunk (98.80 cm) on both inter-row sides of each poplar; (5) 8–4, removing the root system at a distance of eight times the DBH from the trunk (98.80 cm) on four sides of each poplar; (6) 10–2, removing the root system at a distance of ten times the DBH from the trunk (123.50 cm) on both inter-row sides of each poplar; and (7) 10–4, removing the root system at a distance of ten times the DBH from the trunk (123.50 cm) on four sides of each poplar. Each root system was cut with a sharp metal spade to a depth of 30 cm and root pruning was conducted in a line that was either parallel or vertical to the tree row. The treated trees were managed in accordance with routine methods.

### Data collection and determination

For all treatments, mature leaves were sampled during late May, 2015 (the start of the fast-growth period); mid-October, 2015 (the end of the fast-growth period); late May, 2016; mid-October, 2016; late May, 2017; and mid-October, 2017. At each sample date, nine representative samples from every tree were collected for each treatment; the samples were selected from mature leaves on the sunny side of each tree. A portable gas exchange system (LI-6400, LI-COR, Nebraska, US) was used to determine the net photosynthetic rate (*P*_n_), transpiration rate (*T*_r_), and stomatal conductance (*g*_s_) from 09:00 h to 11:00 h. Instantaneous leaf WUE was calculated as the ratio of *P*_n_ to *T*_r_ (*P*_n_/*T*_r_) according to Wong et al. [20] and Ren et al. [21]. The leaves sampled were quickly taken back to the lab, which was placed in an oven at 105°C for 15 min, then dried at 80°C and ground through a 30-mesh screen for the determination of N, P, and K concentrations. Leaf N concentration was measured using the H_2_SO_4_-H_2_O_2_- distillation method, P concentration using the vanadium molybdenum yellow colorimetric method, and K concentration using flame photometric method [19].

Tree height and DBH were measured by the tangent method [22] using a ruler with 0.5-mm accuracy at the beginning of experiment (April 18^th^, 2015) and at the end of the short rotation period (October 18^th^, 2017). The tree volume was calculated using Eq 1:

$$V=3.14d^{{}^{2}}hf/4(f=0.42)$$ (1)

where d and h represent DBH (cm) and tree height (m), respectively [4]. Then the average growth rates of DBH, tree height, and tree volume were calculated according to the method of Meng [23].

Additionally, all the fine roots less than 2 mm diameter in the root distribution zone with the depth of 0–40 cm for all treatments were collected from the soil at the last sample time (mid-October, 2017), according to the method of Du et al. [24]. After scanning, they were processed with WinRHIZO (Regents Instruments Inc. Quebec, Canada) to obtain surface area. Then, fine roots were dried in an oven at 80°C for 48 hours and weighed.

### Statistical analysis

**Fig 1:**
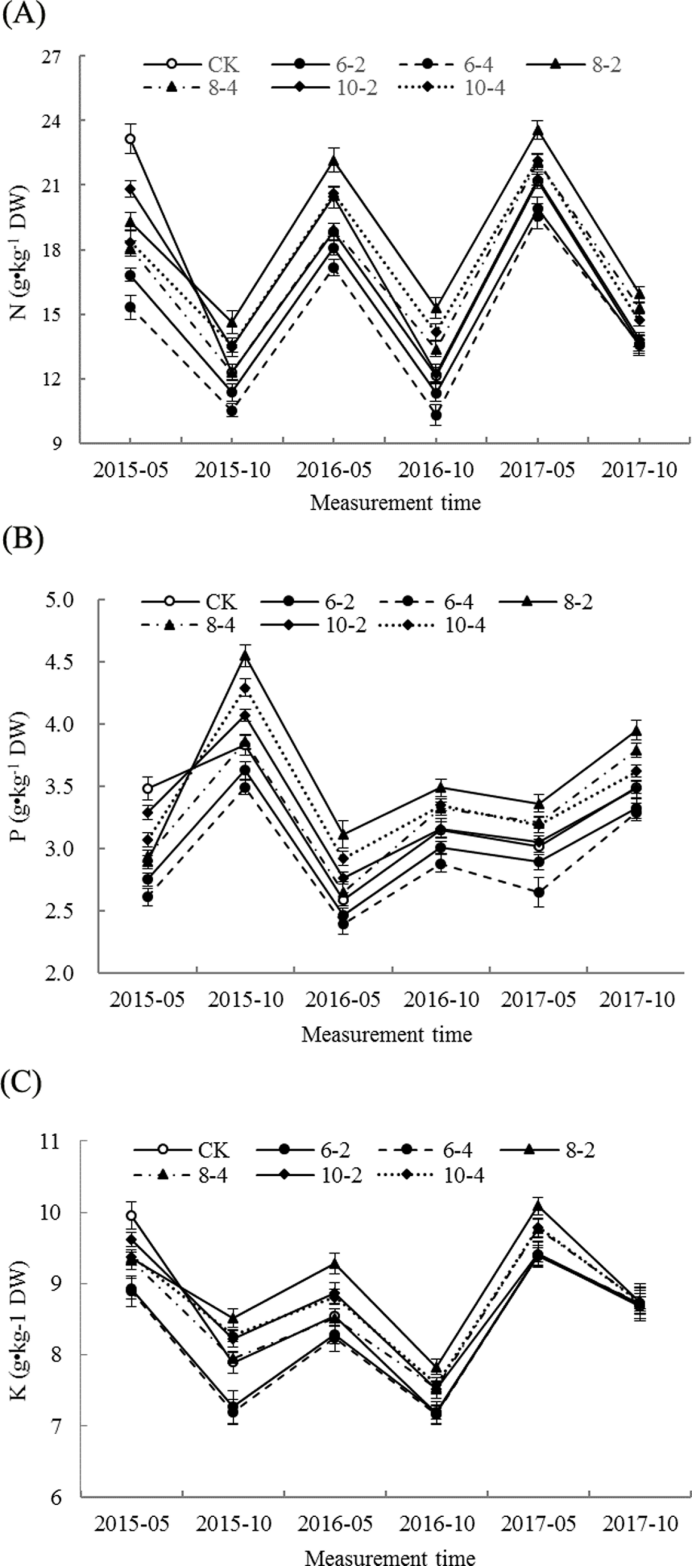
Effects of root pruning on N, P and K concentrations in poplar leaves. Bars are means and error bars are standard deviations (n = 3). (A) N concentration in poplar leaves. (B) P concentration in poplar leaves. (C) K concentration in poplar leaves.

**Fig 2:**
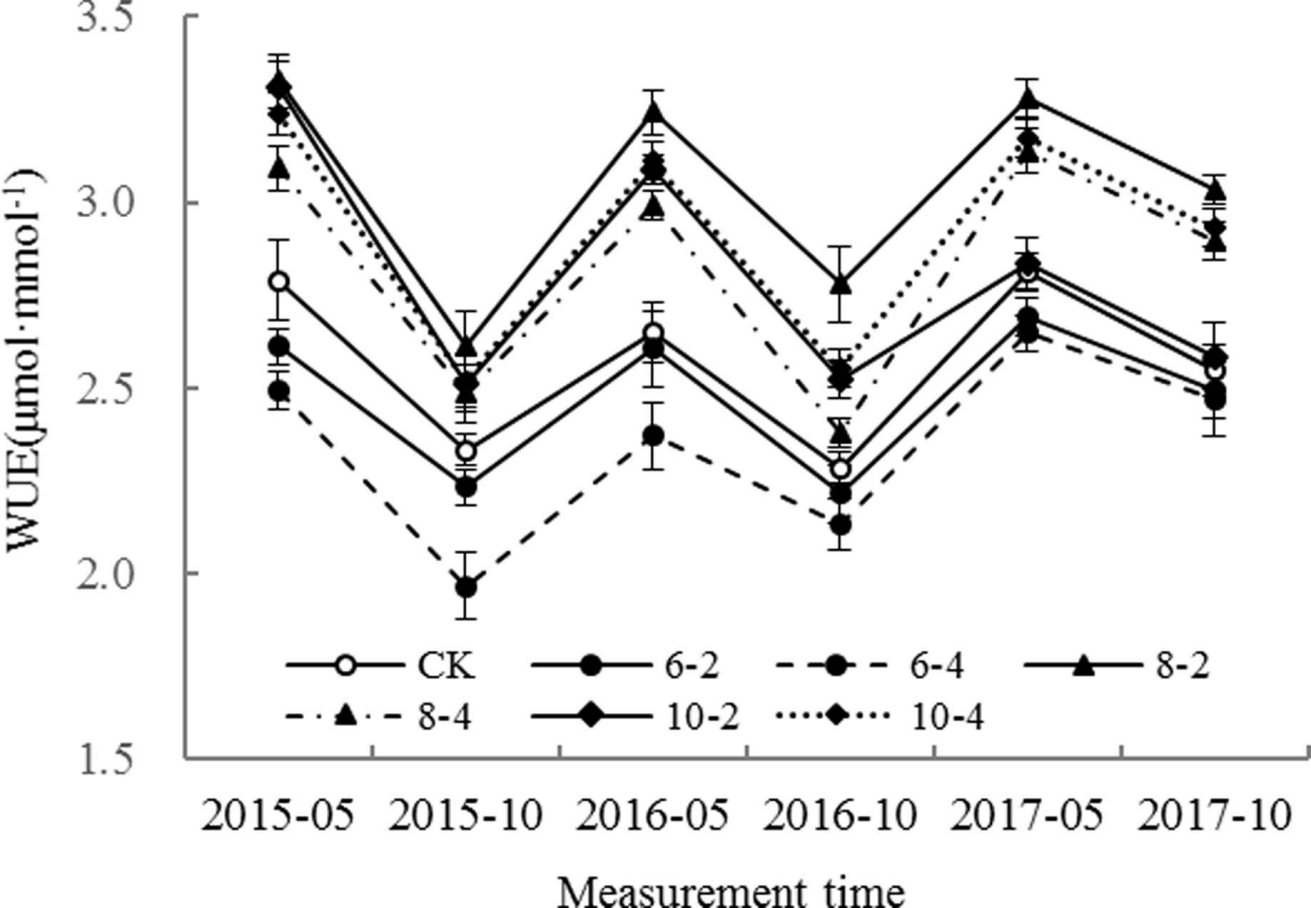
Effects of root pruning on WUE of poplar leaves. Bars are means and error bars are standard deviations (n = 3).

**Fig 3:**
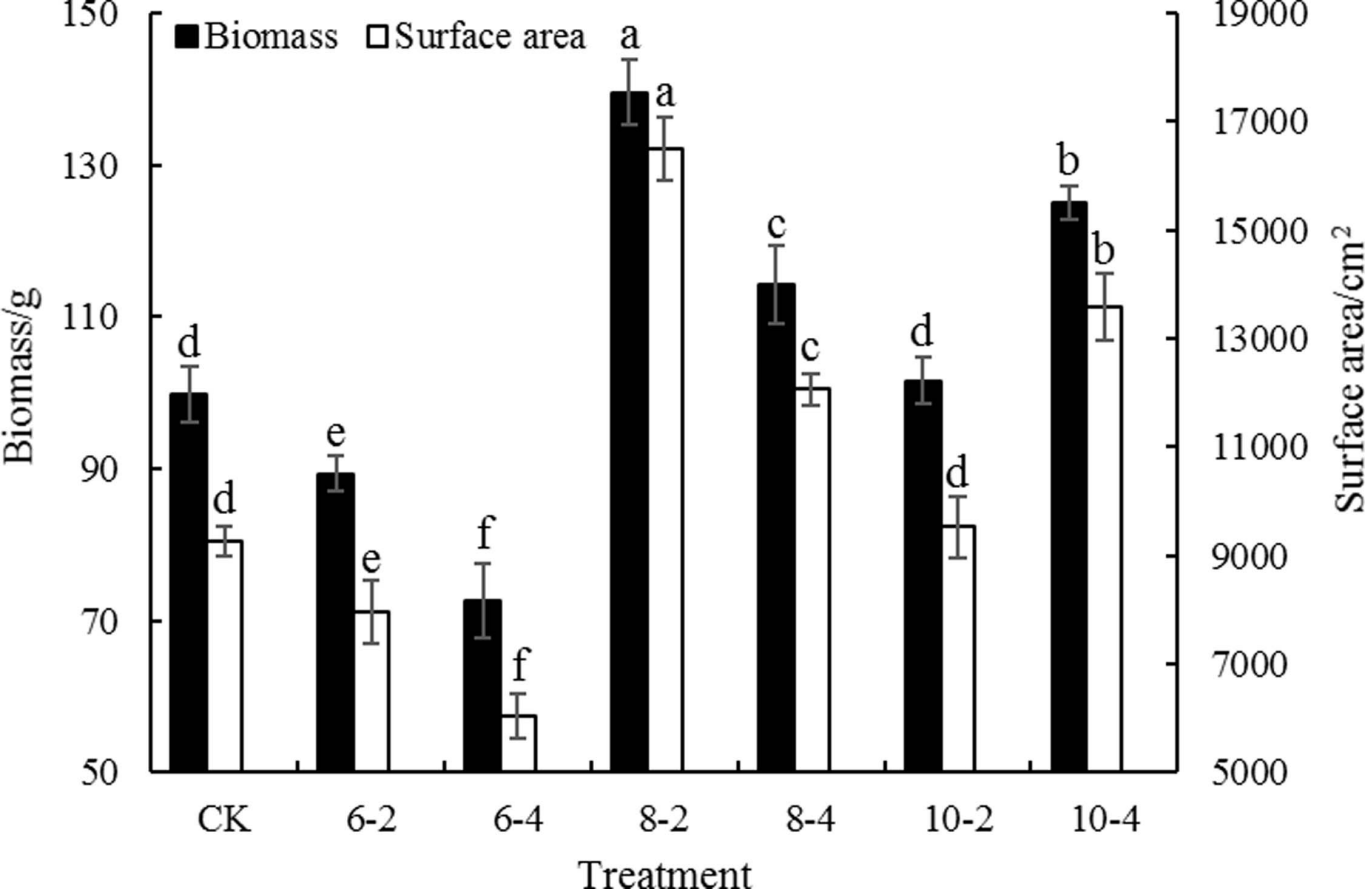
Biomass and surface area of fine root as affected by different pruning treatments. Bars are means and error bars are standard deviations (n = 3). Different letters indicate significant differences among treatments at P<0.05 by LSD.

The data were analyzed as a completely randomized design. An analysis of variance (ANOVA) was carried out to evaluate the effects of root-pruning treatment on leaf nutrients, photosynthetic characteristics, WUE, and growth of poplar at each measurement date. When the ANOVA analysis revealed significant differences between treatments, the least significant difference (LSD) test was conducted to detect differences between individual treatment-level means. All statistical analyses were performed at a significant level of *P* < 0.05. ANOVA and multiple comparisons were performed using SPSS software (version 22.0; SPSS Inc., Chicago, IL, USA). All results in Figs 1–3 and Tables 1–4 are given as the means of three replicates.

**Table 1:** Effects of root pruning on photosynthetic rate of poplar leaves (mean ± SD).

Treatment	Net photosynthetic rate (μmol·m^−2^·s^−1^)					
	2015–05	2015–10	2016–05	2016–10	2017–05	2017–10
**CK**	11.49±0.58a	7.02±0.29c	11.26±0.51d	7.47±0.35cd	11.78±0.37c	7.82±0.29cd
**6–2**	6.97±0.52d	6.45±0.33c	10.18±0.46e	6.84±0.28de	10.93±0.41d	7.21±0.27de
**6–4**	6.46±0.47d	5.62±0.40d	9.29±0.37f	6.25±0.31e	10.85±0.45d	7.18±0.32e
**8–2**	9.61±0.39b	8.86±0.32a	14.81±0.43a	9.89±0.65a	15.39±0.55a	10.22±0.41a
**8–4**	8.75±0.36c	7.65±0.31b	12.68±0.35c	7.96±0.33bc	13.87±0.49b	9.35±0.41b
**10–2**	10.22±0.42b	8.19±0.33b	13.52±0.42b	8.02±0.42bc	12.05±0.38c	7.96±0.39c
10–4	9.81±0.55b	8.23±0.41b	13.59±0.41b	8.57±0.38b	13.96±0.57b	9.29±0.42b

Note: Different letters indicate significant differences among treatments at *P*<0.05 by LSD.

**Table 2:** Effects of root pruning on transpiration rate of poplar leaves (mean ± SD).

Treatment	Transpiration rate (mmol·m^−2^·s^−1^)					
	2015–05	2015–10	2016–05	2016–10	2017–05	2017–10
**CK**	4.12±0.18a	3.01±0.05c	4.25±0.06b	3.27±0.06bc	4.19±0.05cd	3.07±0.07d
**6–2**	2.67±0.05ef	2.89±0.06d	3.91±0.12c	3.09±0.06d	4.06±0.08d	2.89±0.03e
**6–4**	2.59±0.06f	2.86±0.06d	3.92±0.09c	2.93±0.07e	4.10±0.09d	2.91±0.06e
**8–2**	2.89±0.09cd	3.39±0.05a	4.57±0.08a	3.56±0.07a	4.69±0.12a	3.37±0.07a
**8–4**	2.83±0.07de	3.08±0.04c	4.24±0.05b	3.35±0.08b	4.42±0.08b	3.23±0.02b
**10–2**	3.09±0.07b	3.26±0.02b	4.38±0.08b	3.18±0.10cd	4.25±0.05c	3.08±0.05cd
**10–4**	3.03±0.08bc	3.27±0.05b	4.37±0.07b	3.36±0.06b	4.40±0.07b	3.17±0.05bc

Note: Different letters indicate significant differences among treatments at *P*<0.05 by LSD.

**Table 3:** Effects of root pruning on stomatal conductance of poplar leaves (mean ± SD).

Treatment	Stomatal conductance (mmol·m^−2^·s^−1^)					
	2015–05	2015–10	2016–05	2016–10	2017–05	2017–10
**CK**	162±5a	85±7c	168±9c	105±6de	190±9c	118±5c
**6–2**	86±7e	70±9d	149±7d	89±7e	163±8d	96±10d
**6–4**	83±9e	68±6d	117±12e	71±9f	159±10d	93±13d
**8–2**	119±8c	122±7a	236±11a	159±5a	268±12a	166±9a
**8–4**	103±6d	102±6b	176±10c	123±15bc	239±9b	145±8b
**10–2**	144±10b	118±9a	212±9b	121±12cd	198±14c	121±12c
**10–4**	137±8b	103±5b	208±7b	140±10b	230±8b	148±7b

Note: Different letters indicate significant differences among treatments at *P*<0.05 by LSD.

**Table 4:** Effects of root pruning on growth rates of DBH, height and volume (mean ± SD).

Treatment	DBH/cm		Tree height/m		Volume (×10^−2^)/m^3^		DBH growth rate/%	Tree height growth rate/%	Volume growth rate/%
	2015–05	2017–10	2015–05	2017–10	2015–05	2017–10			
**CK**	10.82±0.27a	15.92±0.16d	12.68±0.15a	18.03±0.15b	4.89±0.31a	15.07±0.42d	12.72±0.61d	11.61±0.33d	33.97±0.86d
**6–2**	11.09±0.15a	15.28±0.24e	12.52±0.18a	17.69±0.10c	5.08±0.26a	13.62±0.53e	10.59±0.26e	11.41±0.27d	30.46±0.58e
**6–4**	10.96±0.20a	14.51±0.40f	11.29±0.80a	15.02±0.25e	4.47±0.85a	10.43±0.71f	9.29±0.30f	9.45±0.39e	26.65±0.89f
**8–2**	11.21±0.18a	19.89±0.16a	12.17±0.12a	19.96±0.58a	5.04±0.20a	26.03±0.65a	18.61±0.58a	16.16±0.75a	45.03±0.81a
**8–4**	11.33±0.25a	17.42±0.72c	12.25±0.38a	18.19±0.14b	5.18±0.29a	18.20±0.37c	14.12±0.36c	13.01±0.64c	37.10±1.02c
**10–2**	10.81±0.29a	15.88±0.35d	11.96±0.76a	17.13±0.21d	4.61±0.65a	14.24±0.59de	12.66±0.52d	11.85±0.22d	34.07±0.55d
**10–4**	12.02±0.96a	19.47±0.21b	12.85±0.82a	19.89±0.68a	6.12±0.93a	24.86±0.77b	15.77±0.65b	14.34±0.28b	40.32±0.63b

Note: Different letters indicate significant differences among treatments at *P*<0.05 by LSD.

## Results

### N, P, K concentrations in the leaves

The effects of different treatments on the N, P and K concentrations in the poplar leaves are shown in Fig 1. During late May 2015, 30 days after root pruning, the leaf N, P and K concentrations significantly decreased following root pruning compared with CK, the largest decline occurring in treatments 6–2 and 6–4 followed by 8–2 and 8–4, whereas the smallest decline occurred in the 10–2 and 10–4 treatments. The N and P concentrations in the 8–2 treatment were significantly increased over CK, and were also higher compared with the other treatments at the subsequent survey dates. Similarly, the K concentration in the 8–2 treatment also increased between the 2^nd^ and 5^th^ survey dates. However, the N and P concentrations in the 8–4 treatment were not significantly different from CK at the 2^nd^ and 3^rd^ survey dates, but were significantly increased compared with CK in the following survey. The 10–2 treatment increased the N and P concentrations compared with CK at the 2^nd^ and 3^rd^ survey dates, but did not differ significantly from CK from the 4^th^ to 6^th^ survey dates. However, the K concentration was significantly higher than that of CK from the 2^nd^ to 4^th^ survey dates. The N, P, and K concentrations in the 10–4 treatment were higher compared with CK from the 2^nd^ to 5^th^ survey dates, whereas the N, P and K concentrations in the 6–2 and 6–4 treatments were significantly decreased compared with CK, with the lowest concentrations occurring in the 6–4 treatment. The results indicated that different root-pruning treatments had varied effects on the concentrations of N, P and K in poplar leaves. Among all root-pruning treatments, the 8–2 treatment had more positive effects on the nutritional status of poplar tree leaves.

### Leaf photosynthesis

As shown in Tables 1–3, the net photosynthetic rate (*P*_n_), transpiration rate (*T*_r_), and stomatal conductance (*g*_s_) in poplar were significantly decreased compared with CK 1 month after root pruning, with the largest decline occurring in the 6–4 treatment and the smallest in the 10–2 treatment. *P*_*n*_ and *g*_*s*_ showed consistent seasonal variation and were significantly higher in the 8–2 treatment than in the other treatments. Compared with CK, root pruning in the 8–4 and 10–4 treatments enhanced *P*_*n*_ and *g*_*s*_, whereas these were decreased in the 6–2 and 6–4 treatments, especially in the latter. As shown in Table 2, *T*_*r*_ was also the highest in the 8–2 treatment, and T_*r*_ in the 8–4 treatment was significantly increased compared with CK in the 3^rd^ year after root pruning (i.e, in 2017). Even though *T*_*r*_ in the 10–2 treatment was significantly higher than in CK at the 2^nd^ and 3^rd^ survey dates, no differences were found in the following survey dates. The aforementioned results showed that root pruning had significant effects on the photosynthetic characteristics of poplar leaves, which was closely related to the severity of root pruning.

### WUE of leaves

As shown in Fig 2, the WUE of leaves in the 8–2, 8–4, 10–2 and 10–4 treatments significantly increased over CK 1 month after root pruning, whereas it was significantly decreased in the 6–2 and 6–4 treatments. The largest decline in WUE occurred in the 6–4 treatment. In subsequent survey dates, the WUE among all treatments was highest in the 8–2 treatment. Compared with CK, WUE was significantly higher in the 10–4 and 8–4 treatments. There was no difference in WUE between the 10–2 treatment and CK in the 3^rd^ year after root pruning. However, leaf WUE in the 6–2 and 6–4 treatments was lower compared with CK within 3 years after root pruning, with the lowest value being found in the 6–4 treatment. Although there was an obvious regulatory effect of root pruning on the WUE of poplar leaves, not all root pruning treatments improved WUE.

### Growth rates of DBH, tree height, and volume

The effects of root pruning on growth rates of DBH, height, and volume of poplar trees in over consecutive years are detailed in Table 4. The average growth rates of DBH, tree height, and tree volume showed consistent variation within treatments, with the overall order of measurements as follows: 8–2>10–4>8–4>10–2≈CK>6–2>6–4. For average growth rates of DBH, tree height, and volume, the highest values all occurred in the 8–2 treatment, increasing by 46.31%, 39.19%, and 32.56%, respectively, compared with CK. However, the average growth rates of DBH and volume in the 6–4 treatment were lower than those in CK. Therefore, different root pruning treatments had significantly different effects on poplar growth.

### Discussion

*Populus* × *euramericana* cv. ‘Neva’ is one of the most suitable species for afforestation and woody production in arid and semiarid areas of China [5]. The successful establishment and rapid growth of the trees depend on the fast growth and nutrient-absorbing ability of the root system. Previous preliminary studies on winter jujube and poplar illustrated that different intensities of root pruning have disparate effects on tree nutrition [4, 12]; however, these studies only pruned the roots on two sides of each tree. In the current study, comprehensive methods of root pruning were implemented by combining three intensities (six, eight, or ten times the DBH from the trunk) and two orientations (two or four sides). The results showed that the N, P and K concentrations of leaves in all treatments decreased in the short term (30 days) following root pruning. This could be because some of the absorptive roots of the trees were removed by pruning, thereby significantly weakening the absorptive capacity of the root system and resulting in a rapid decrease in nutrient uptake [9]. On subsequent survey dates, the N and P concentrations of leaves in the 8–2 treatment had increased and were significantly higher compared with other treatments, which was in part agreement with a previous study [4]. Compared with the 8–4 treatment, the same strength of root pruning on two sides of the tree in the 8–2 treatment not only stimulated the growth of new roots [5], but also increased the absorption area of the roots (Fig 3), which subsequently increased the nutrient uptake [9, 25].

In the 8–4 and 10–4 treatments, root pruning was conducted on four sides; thus, the damage to the roots was much bigger. However, the nutrient concentrations of leaves still significantly increased compared with those in CK during the later stages of the experiment. This might be because, even though the increase in poplar growth had a dilution effect on nutrient concentrations of the leaves, a large number of new roots gradually sprouted on the root incisions on all four sides of the trees over time and absorbed a large amount of nutrients from soil, resulting in the increase in leaf nutrient concentrations in these treatments (Figs 1 and 3). This also suggests that new roots growing from root incisions were able to strongly absorb soil nutrients. Root pruning in the 10–2 treatment was carried out relatively far from the trunk, which inflicted less damage to the root system and shortened the recovery time of the root; thus, the nutrient content of the leaves increased rapidly compared with CK [4, 26]. However, because the stimulation effect of root pruning on the incision was significantly reduced, the amount of new roots and the root absorption area were less compared with other treatments [25, 27]; thus, the incremental change in nutrient content in the leaves was not significant during the later stages of the study. However, in the 6–2 and 6–4 treatments, root pruning occurred closest to the trunk, which resulted in the most damage to the root system, especially in the 6–4 treatment. This was also possibly correlated with the fact that, because the roots were shorter following pruning, the volume of soil the trees had access to was limited. Thus, the root recovery time was prolonged and their nutrient uptake would have lagged behind that in other treatments. This suggested that the intensity and orientation of root pruning have a decisive role in the leaf nutrition of poplar trees.

Plants are not able to absorb as much water and nutrients within a certain period of time after root pruning, mainly because the latter initially decreases the root surface area. With an increase in root-pruning intensity, the net photosynthetic rate, transpiration rate, and stomatal conductance correspondingly decrease [11–12]. Similar conclusions were drawn from the results from the first survey date in the current study. It might be that roots and leaves are able to communicate via signaling molecules [28] that might result in partial stomatal closure and, thus, a decrease in stomatal conductance and transpiration rate, in leaves when the roots have been fully or partially removed [29]. Root pruning caused an initial decrease in the transpiration rate, which was the direct cause of the decrease in the net photosynthetic rate in the current study. However, with the emergence of new roots, an increase in photosynthetic parameters was observed for the 8–2, 8–4, 10–2, and 10–4 treatments from the 2^nd^ to 5^th^ survey dates compared with CK. These findings are consistent with those obtained in previous studies [12, 29].

The ratio of photosynthesis to transpiration is an indicator of the water use efficiency of leaves [15–16, 21]. The experimental results showed that *g*_s_ in the 8–2, 8–4, 10–2 and 10–4 treatments was decreased at 30 days after root pruning, as were the net photosynthetic rate and transpiration rate. In these treatments, the leaf transpiration rate decreased faster than the net photosynthetic rate_,_ so that WUE increased, and was significantly higher than that of CK. This might be because the decline in stomatal conductance had less influence on photosynthesis than it had on transpiration [12]. Transpiration rate has a strong dependence on the stoma, thus partial stomatal closure would have been conducive to improving the WUE of the leaves. On the subsequent survey dates, the net photosynthetic rate, transpiration rate, and stomatal conductance in the 8–2, 8–4 and 10–4 treatments were all higher than those of CK. In these treatments, the WUE was also higher compared with CK, probably because of a faster increase in net photosynthetic rate than transpiration rate, along with the increase in stomatal conductance as a result of root pruning. High WUE is beneficial for enhancing the total production of plants under variable soil water content as an adaptation to water shortage [2]. However, the current results indicated that WUE in the 6–2 and 6–4 treatments was lower than CK during the 3 years after root pruning, especially in the 6–4 treatment, suggesting that excessive root pruning would reduce the WUE because of irrecoverable damage to the root system.

In addition, the average growth rate of the tree volume in the 8–2 treatment was the highest among all treatments, which could be related to the improved leaf nutrient content and higher photosynthetic productivity and WUE of the leaves. Also, the 10–4 and 8–4 treatments increased the growth rate of tree volume compared with CK, which indicated that root pruning on all four sides resulted in significant harm to the trees initially, but possibly increased the nutrient absorption capacity of the root system at later survey dates. No significant differences in volume growth rate were found between the 10–2 treatment and CK. However, both the 6–2 and 6–4 treatments decreased the growth rate of tree volume compared with CK. These results further confirmed that the strength and orientation of root pruning were of great importance for regulating the growth of poplar trees. Furthermore, the experimental area is windy in spring, but the wind is usually no stronger than force 6 (strong breeze); thus, the pruned trees never encountered the problem of windthrow.

## Conclusions

In conclusion, it is evident from this study that the application of root pruning had significant regulation effects on the nutrient content, photosynthetic characteristics, and WUE of poplar leaves, as well as on tree growth at the stage of canopy closing during the later stages of a short rotation. The selection of root-pruning intensity and orientation plays a decisive role in the effectiveness of this technique for the management of poplar plantations.
